# Crystal structure of 3-[2-(1,3-thia­zol-2-yl)diazen-1-yl]pyridine-2,6-di­amine monohydrate

**DOI:** 10.1107/S2056989018004693

**Published:** 2018-03-27

**Authors:** Ratanon Chotima, Bussaba Boonseng, Akkharadet Piyasaengthong, Apisit Songsasen, Kittipong Chainok

**Affiliations:** a Department of Chemistry, Faculty of Science, Naresuan University, Muang, Phitsanulok 65000, Thailand; b Department of Chemistry, Faculty of Science, Kasetsart University, Chatuchak, Bangkok 10900, Thailand; c Materials and Textile Technology, Faculty of Science and Technology, Thammasat University, Khlong Luang, Pathum Thani 12121, Thailand

**Keywords:** crystal structure, azo dyes, hydrogen bonding, π–π stacking

## Abstract

In the title hydrated azo compound, C_8_H_8_N_6_S·H_2_O, the two aromatic groups are close to coplanar with the dihedral angle between the mean planes of the thia­zole and pyridine rings being 2.9 (2)°. The organic mol­ecule adopts an *E* configuration with respect to the double bond of the azo bridge. In the crystal, mol­ecules are linked by (amine)N—H⋯N(pyridine), (amine)N—H⋯O(water) and (water)O—H⋯N(thia­zole) hydrogen bonds along with π–π inter­actions involving pairs of thia­zole rings and pairs of pyridine rings. The plane-to-plane distance between two parallel mol­ecules is 3.7856 (4) Å and corresponds to the length of the *a* axis. In this way, a layer structure parallel to (010) is formed. The layers are linked by weak C—H⋯S hydrogen bonds, eventually resulting in a three-dimensional network.

## Abstract 2

The organic mol­ecule in the title hydrate shows an *E* configuration with respect to the azo functionality.

## Chemical context   

Azo compounds are one of the most important organic dyes used in industrial applications to colour various consumer goods such as leather, plastics and cosmetics (Kaur *et al.*, 2018 ▸). The main characteristic of these compounds is the chromophore of the azo group (–N=N–), which is responsible for the color of the dyes. Compounds with an aromatic thia­zolylazo moiety are a subclass of azo dyes, which contain the thia­zole group on one side of the azo linkage and are important ligands in coordination chemistry (Kaim, 2001 ▸). In this regard, zinc complexes with polydentate chelating thia­zolylazo ligands have been prepared as luminescence probes for selectively sensing phosphates (Hens *et al.*, 2015 ▸). Recently, Piyasaengthong *et al.* (2015 ▸) reported the synthesis of a gold(III) complex of 3-(2′-thia­zolylazo)-2,6-di­amino­pyridine and investigated its pepsin inhibition.
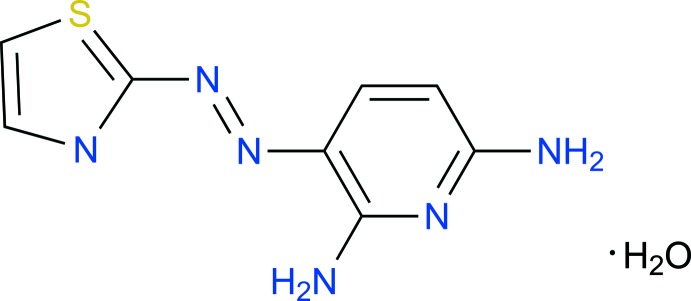


We report here the crystal structure of 3-(2′-thia­zolylazo)-2,6-di­amino­pyridine monohydrate, C_8_H_8_N_6_S·H_2_O, (I), obtained through the diazo­tization of 2-amino­thia­zole followed by a coupling reaction with 2,6-di­amino­pyridine (Montelongo *et al.*, 1982 ▸).

## Structural commentary   

**Figure 1:**
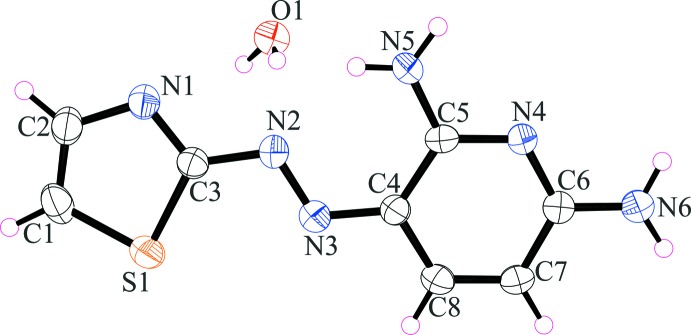
The mol­ecular structure of the organic entity and the water mol­ecule in compound (I), with the atom labelling and displacement ellipsoids drawn at the 50% probability level.

The mol­ecular entities of (I) with atom labelling are presented in Fig. 1 ▸. The organic mol­ecule has an *E* configuration with respect to the azo bridge (–N2=N3–), and is essentially planar with an r.m.s deviation of the fitted non-hydrogen atoms being 0.033 Å. The amine N5 and N6 atoms are 0.044 (2) and −0.059 (3) Å, respectively, out of this plane. The thia­zole ring (C1–C3, N1, S1) makes a dihedral angle of 2.9 (2)° with the pyridine ring (C4–C8, N4). An intra­molecular N5—H5*A*⋯N2 hydrogen bond is observed (Table 1 ▸), showing an *S*(6) ring motif.

## Supra­molecular features   

**Figure 2:**
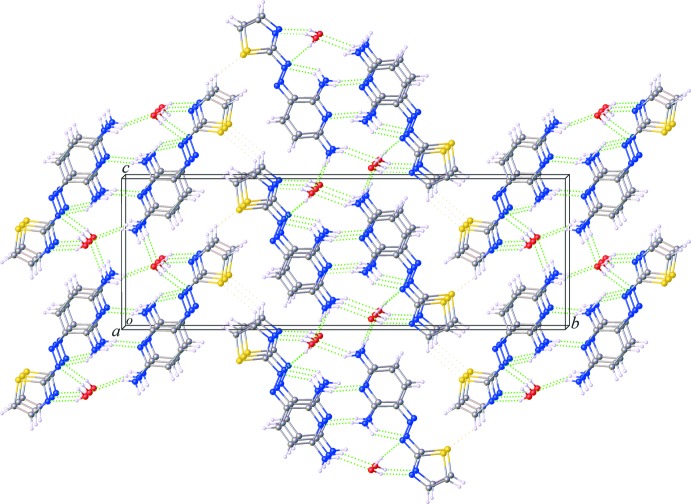
The unit-cell packing in (I) , viewed approximately down [100]. The classical O—H⋯N, and N—H⋯O hydrogen bonds are shown as green dashed lines (see Table 1 ▸ for numerical details).

In the crystal of (I), extensive (amine)N—H⋯N(pyridine), (amine)N—H⋯O(water) and (water)O—H⋯N(thia­zole) hydrogen bonds (Table 1 ▸) are present. Together with π–π inter­actions involving pairs of thia­zole rings and pairs of pyridine rings with a plane-to-plane distance between two parallel mol­ecules of 3.7856 (4) Å, a layered structure parallel to the *ac* plane is formed (Fig. 2 ▸). Weak C—H⋯S hydrogen bonds between adjacent thia­zole rings further consolidate the crystal packing, thus generating a three-dimensional network.

## Database survey   

A search of the Cambridge Structural Database (Groom *et al.*, 2016 ▸) for compounds with the (*E*)-2-(pyridin-3-yldiazen­yl)thia­zole moiety gave no hits. However, structures of substituted thia­zolylazo derivatives were found, for example, 5-(di­ethyl­amino)-2-(2-thia­zolylazo)phenol (QAVNAD; Zhang *et al.*, 2005 ▸), 4-(2′-thia­zolylazo)pyrocatechol (TZAZPC; Apinitis, 1978 ▸), 1-(2-thia­zolylazo)-6-bromo-2-naphthol (TAZBRN10; Kurahashi *et al.*, 1976 ▸) and 1-(2-thia­zolylazo)-2-naphthol (TAZNPL10; Kurahashi, 1976 ▸).

## Synthesis and crystallization   

2-Amino­thia­zole (1.0 g, 0.009 mol) was dissolved in 6 *M* hydro­chloric acid (16 ml) with sodium nitrite (0.7 g, 0.01 mol). The mixture was stirred at a temperature between 268 and 273 K while a solution of 2,6-di­amino­pyridine (1.0 g, 0.009 mol) in 40 ml of 4 *M* hydro­chloric acid was added. The reaction mixture was stirred for 1 h and then adjusted to pH 6.0 by 0.001 *M* sodium hydroxide. The red precipitate formed was filtered through suction and washed with water. Suitable crystals for X-ray analysis were grown by recrystallization using the vapor diffusion technique in a methanol-hexane mixture at 253 K [yield 1.12 g, 51%]. ^1^H NMR (400 MHz, 298 K, C_2_D_6_OS): *δ* 6.10 (*d*, *m*-Ar*H* py, 1H), 7.39 (*d*, thia­zole-*H*, 1H), 7.55 (*d*, *p*-Ar*H* py, 1H), 7.703 (*d*, thia­zole-*H*, 1H). Mass spec. (ESI) *m*/*z* 220.9 (*M* ^+^), 136.2, 108.3, 81.4. IR–KBr (cm^−1^): 3335 (*w*), 3217 (*w*), 3082 (*w*), 1660 (*s*), 1631 (*s*), 1454 (*m*), 1292 (*s*), 1159 (m). Analysis calculated for C_8_H_10_N_6_OS: C, 43.64; H, 3.66; N, 38.16; 14.56. Found: C, 43.80; H, 3.79; N, 38.45; S,14.78.

## Refinement   

Crystal data, data collection and structure refinement details are summarized in Table 2 ▸. H atoms bonded to O and N atoms were located in difference-Fourier maps and refined with distance restraints of 0.84±0.02 Å with *U*_iso_(H) = 1.5*U*_eq_(O) and 0.86±0.02 Å with *U*_iso_(H) = 1.2*U*_eq_(N), respectively. The C-bound H atoms were included in calculated positions and treated as riding atoms: C—H = 0.93 Å with *U*_iso_(H) = 1.2*U*_eq_(C).

## Supplementary Material

Crystal structure: contains datablock(s) I. DOI: 10.1107/S2056989018004693/wm5438sup1.cif

Structure factors: contains datablock(s) I. DOI: 10.1107/S2056989018004693/wm5438Isup2.hkl

Supporting information file. DOI: 10.1107/S2056989018004693/wm5438Isup3.cdx

Supporting information file. DOI: 10.1107/S2056989018004693/wm5438Isup4.cml

CCDC reference: 1831600

Additional supporting information:  crystallographic information; 3D view; checkCIF report

## Figures and Tables

**Table 1:** Hydrogen-bond geometry (Å, °)

*D*—H⋯*A*	*D*—H	H⋯*A*	*D*⋯*A*	*D*—H⋯*A*
C2—H2⋯S1^i^	0.93	3.02	3.665 (3)	128
N5—H5*A*⋯N2	0.86 (1)	2.03 (3)	2.645 (4)	128 (3)
N5—H5*A*⋯O1	0.86 (1)	2.59 (2)	3.314 (4)	143 (3)
N5—H5*B*⋯N4^ii^	0.86 (1)	2.14 (1)	2.998 (4)	172 (3)
N6—H6*A*⋯O1^ii^	0.86 (1)	2.27 (2)	3.048 (4)	151 (4)
N6—H6*B*⋯O1^iii^	0.86 (1)	2.13 (1)	2.988 (4)	177 (4)
O1—H1*A*⋯N1	0.84 (1)	2.13 (3)	2.923 (4)	158 (6)
O1—H1*B*⋯N2^iv^	0.84 (1)	2.31 (2)	3.143 (4)	170 (9)

Symmetry codes: (i) 
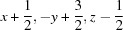
; (ii) 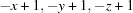; (iii) 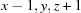; (iv) 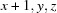.

**Table 2:** Experimental details

Crystal data	
Chemical formula	C_8_H_8_N_6_S·H_2_O
*M*_r_	238.28
Crystal system, space group	Monoclinic, *P*2_1_/*n*
Temperature (K)	296
*a*, *b*, *c* (Å)	3.7856 (4), 28.393 (3), 9.6324 (9)
β (°)	93.824 (3)
*V* (Å^3^)	1033.02 (17)
*Z*	4
Radiation type	Mo *K*α
μ (mm^−1^)	0.30
Crystal size (mm)	0.28 × 0.08 × 0.04

Data collection	
Diffractometer	Bruker D8 QUEST CMOS
Absorption correction	Multi-scan (*SADABS*; Bruker, 2016 ▸)
*T*_min_, *T*_max_	0.644, 0.745
No. of measured, independent and observed [*I* > 2σ(*I*)] reflections	11837, 2100, 1670
*R*_int_	0.054
(sin θ/λ)_max_ (Å^−1^)	0.630

Refinement	
*R*[*F* ^2^ > 2σ(*F* ^2^)], *wR*(*F* ^2^), *S*	0.061, 0.121, 1.17
No. of reflections	2100
No. of parameters	169
No. of restraints	6
H-atom treatment	H atoms treated by a mixture of independent and constrained refinement
Δρ_max_, Δρ_min_ (e Å^−3^)	0.33, −0.26

Computer programs: *APEX3* and *SAINT* (Bruker, 2016 ▸), *SHELXT* (Sheldrick, 2015*a* ▸), *SHELXL2014* (Sheldrick, 2015*b* ▸) and *OLEX2* (Dolomanov *et al.*, 2009 ▸).

## supplementary crystallographic information

### Crystal data

**Table:**

C_8_H_8_N_6_S·H_2_O	*F*(000) = 496
*M**_r_* = 238.28	*D*_x_ = 1.532 Mg m^−^^3^
Monoclinic, *P*2_1_/*n*	Mo *K*α radiation, λ = 0.71073 Å
*a* = 3.7856 (4) Å	Cell parameters from 4156 reflections
*b* = 28.393 (3) Å	θ = 3.0–26.6°
*c* = 9.6324 (9) Å	µ = 0.30 mm^−^^1^
β = 93.824 (3)°	*T* = 296 K
*V* = 1033.02 (17) Å^3^	Needle, dark orange
*Z* = 4	0.28 × 0.08 × 0.04 mm

### Data collection

**Table:**

Bruker D8 QUEST CMOS diffractometer	2100 independent reflections
Radiation source: microfocus sealed x-ray tube, Incoatec Iµus	1670 reflections with *I* > 2σ(*I*)
GraphiteDouble Bounce Multilayer Mirror monochromator	*R*_int_ = 0.054
Detector resolution: 10.5 pixels mm^-1^	θ_max_ = 26.6°, θ_min_ = 3.6°
ω and φ scans	*h* = −4→4
Absorption correction: multi-scan (*SADABS*; Bruker, 2016)	*k* = −35→35
*T*_min_ = 0.644, *T*_max_ = 0.745	*l* = −12→11
11837 measured reflections	

### Refinement

**Table:**

Refinement on *F*^2^	Primary atom site location: dual
Least-squares matrix: full	Hydrogen site location: mixed
*R*[*F*^2^ > 2σ(*F*^2^)] = 0.061	H atoms treated by a mixture of independent and constrained refinement
*wR*(*F*^2^) = 0.121	*w* = 1/[σ^2^(*F*_o_^2^) + (0.0131*P*)^2^ + 1.7616*P*] where *P* = (*F*_o_^2^ + 2*F*_c_^2^)/3
*S* = 1.17	(Δ/σ)_max_ < 0.001
2100 reflections	Δρ_max_ = 0.33 e Å^−^^3^
169 parameters	Δρ_min_ = −0.26 e Å^−^^3^
6 restraints	

### Special details

**Table:**

Geometry. All esds (except the esd in the dihedral angle between two l.s. planes) are estimated using the full covariance matrix. The cell esds are taken into account individually in the estimation of esds in distances, angles and torsion angles; correlations between esds in cell parameters are only used when they are defined by crystal symmetry. An approximate (isotropic) treatment of cell esds is used for estimating esds involving l.s. planes.

### Fractional atomic coordinates and isotropic or equivalent isotropic displacement parameters (Å^2^)

**Table:**

	*x*	*y*	*z*	*U*_iso_*/*U*_eq_	
S1	0.1445 (2)	0.72666 (3)	0.18385 (9)	0.0377 (2)	
N1	0.4021 (8)	0.65791 (9)	0.0492 (3)	0.0356 (6)	
N2	0.2953 (7)	0.63596 (9)	0.2749 (3)	0.0317 (6)	
N3	0.1805 (7)	0.65155 (9)	0.3905 (3)	0.0310 (6)	
N4	0.2611 (7)	0.54434 (8)	0.6116 (3)	0.0299 (6)	
N5	0.4246 (8)	0.55257 (10)	0.3899 (3)	0.0379 (7)	
N6	0.1041 (9)	0.53404 (10)	0.8343 (3)	0.0437 (8)	
C1	0.2378 (10)	0.73517 (12)	0.0133 (4)	0.0415 (8)	
H1	0.2022	0.7632	−0.0355	0.050*	
C2	0.3691 (10)	0.69560 (12)	−0.0395 (4)	0.0409 (8)	
H2	0.4337	0.6939	−0.1308	0.049*	
C3	0.2931 (8)	0.66920 (10)	0.1706 (3)	0.0303 (7)	
C4	0.1707 (8)	0.62052 (10)	0.4978 (3)	0.0284 (7)	
C5	0.2873 (8)	0.57206 (10)	0.4993 (3)	0.0279 (7)	
C6	0.1248 (8)	0.56226 (10)	0.7246 (3)	0.0306 (7)	
C7	0.0081 (9)	0.60975 (11)	0.7330 (3)	0.0342 (7)	
H7	−0.0845	0.6212	0.8134	0.041*	
C8	0.0359 (8)	0.63760 (11)	0.6209 (3)	0.0335 (7)	
H8	−0.0357	0.6689	0.6250	0.040*	
O1	0.7912 (8)	0.56959 (9)	0.0910 (3)	0.0489 (7)	
H5A	0.471 (9)	0.5698 (10)	0.320 (2)	0.040 (10)*	
H5B	0.504 (9)	0.5242 (6)	0.397 (4)	0.047 (11)*	
H6A	0.166 (10)	0.5050 (5)	0.826 (4)	0.058 (12)*	
H6B	0.022 (10)	0.5438 (12)	0.910 (2)	0.052 (12)*	
H1A	0.647 (12)	0.5905 (15)	0.062 (6)	0.11 (2)*	
H1B	0.936 (19)	0.584 (3)	0.145 (8)	0.23 (5)*	

### Atomic displacement parameters (Å^2^)

**Table:**

	*U*^11^	*U*^22^	*U*^33^	*U*^12^	*U*^13^	*U*^23^
S1	0.0472 (5)	0.0283 (4)	0.0375 (5)	0.0039 (4)	0.0011 (4)	−0.0003 (3)
N1	0.0457 (16)	0.0316 (14)	0.0299 (15)	−0.0034 (12)	0.0044 (13)	−0.0004 (11)
N2	0.0362 (15)	0.0295 (13)	0.0295 (15)	0.0017 (11)	0.0028 (12)	0.0031 (11)
N3	0.0332 (14)	0.0286 (13)	0.0313 (15)	0.0013 (11)	0.0018 (12)	0.0008 (11)
N4	0.0342 (14)	0.0265 (13)	0.0292 (14)	0.0033 (11)	0.0048 (12)	0.0011 (10)
N5	0.0555 (19)	0.0264 (14)	0.0331 (16)	0.0100 (13)	0.0127 (14)	0.0034 (12)
N6	0.064 (2)	0.0359 (16)	0.0331 (17)	0.0112 (15)	0.0151 (15)	0.0028 (13)
C1	0.053 (2)	0.0324 (17)	0.037 (2)	−0.0067 (15)	−0.0083 (17)	0.0076 (14)
C2	0.054 (2)	0.0386 (18)	0.0298 (18)	−0.0102 (16)	0.0034 (16)	0.0041 (14)
C3	0.0321 (17)	0.0261 (15)	0.0323 (18)	−0.0027 (12)	0.0002 (14)	−0.0006 (12)
C4	0.0305 (16)	0.0259 (14)	0.0287 (16)	0.0015 (12)	0.0006 (13)	0.0007 (12)
C5	0.0273 (15)	0.0266 (15)	0.0300 (17)	0.0002 (12)	0.0028 (13)	−0.0027 (12)
C6	0.0309 (16)	0.0309 (16)	0.0301 (17)	0.0002 (13)	0.0033 (14)	0.0002 (12)
C7	0.0392 (18)	0.0327 (16)	0.0314 (18)	0.0054 (14)	0.0082 (15)	−0.0043 (13)
C8	0.0376 (18)	0.0272 (15)	0.0355 (19)	0.0065 (13)	0.0021 (15)	−0.0019 (13)
O1	0.0621 (18)	0.0385 (14)	0.0474 (16)	0.0045 (13)	0.0123 (14)	−0.0006 (12)

### Geometric parameters (Å, º)

**Table:**

S1—C1	1.720 (4)	N6—H6A	0.863 (10)
S1—C3	1.733 (3)	N6—H6B	0.860 (10)
N1—C2	1.370 (4)	C1—H1	0.9300
N1—C3	1.305 (4)	C1—C2	1.342 (5)
N2—N3	1.300 (3)	C2—H2	0.9300
N2—C3	1.379 (4)	C4—C5	1.445 (4)
N3—C4	1.360 (4)	C4—C8	1.408 (4)
N4—C5	1.347 (4)	C6—C7	1.423 (4)
N4—C6	1.336 (4)	C7—H7	0.9300
N5—C5	1.326 (4)	C7—C8	1.348 (4)
N5—H5A	0.858 (10)	C8—H8	0.9300
N5—H5B	0.861 (10)	O1—H1A	0.841 (10)
N6—C6	1.333 (4)	O1—H1B	0.840 (10)
			
C1—S1—C3	88.47 (16)	N1—C3—N2	119.9 (3)
C3—N1—C2	110.2 (3)	N2—C3—S1	125.2 (2)
N3—N2—C3	113.9 (2)	N3—C4—C5	126.9 (3)
N2—N3—C4	117.2 (2)	N3—C4—C8	116.5 (3)
C6—N4—C5	119.0 (2)	C8—C4—C5	116.5 (3)
C5—N5—H5A	120 (2)	N4—C5—C4	121.7 (3)
C5—N5—H5B	119 (2)	N5—C5—N4	116.6 (3)
H5A—N5—H5B	121 (3)	N5—C5—C4	121.7 (3)
C6—N6—H6A	118 (3)	N4—C6—C7	123.0 (3)
C6—N6—H6B	122 (3)	N6—C6—N4	117.6 (3)
H6A—N6—H6B	120 (4)	N6—C6—C7	119.3 (3)
S1—C1—H1	124.8	C6—C7—H7	121.0
C2—C1—S1	110.4 (3)	C8—C7—C6	118.0 (3)
C2—C1—H1	124.8	C8—C7—H7	121.0
N1—C2—H2	122.0	C4—C8—H8	119.2
C1—C2—N1	116.0 (3)	C7—C8—C4	121.6 (3)
C1—C2—H2	122.0	C7—C8—H8	119.2
N1—C3—S1	114.9 (2)	H1A—O1—H1B	104 (7)
			
S1—C1—C2—N1	−0.1 (4)	C2—N1—C3—N2	178.4 (3)
N2—N3—C4—C5	2.5 (5)	C3—S1—C1—C2	0.0 (3)
N2—N3—C4—C8	−178.2 (3)	C3—N1—C2—C1	0.2 (5)
N3—N2—C3—S1	−1.5 (4)	C3—N2—N3—C4	179.2 (3)
N3—N2—C3—N1	−180.0 (3)	C5—N4—C6—N6	−179.6 (3)
N3—C4—C5—N4	−179.3 (3)	C5—N4—C6—C7	−0.7 (5)
N3—C4—C5—N5	0.5 (5)	C5—C4—C8—C7	−1.8 (5)
N3—C4—C8—C7	178.8 (3)	C6—N4—C5—N5	179.9 (3)
N4—C6—C7—C8	0.3 (5)	C6—N4—C5—C4	−0.3 (4)
N6—C6—C7—C8	179.3 (3)	C6—C7—C8—C4	1.0 (5)
C1—S1—C3—N1	0.1 (3)	C8—C4—C5—N4	1.4 (4)
C1—S1—C3—N2	−178.5 (3)	C8—C4—C5—N5	−178.8 (3)
C2—N1—C3—S1	−0.2 (4)		

### Hydrogen-bond geometry (Å, º)

**Table:**

*D*—H···*A*	*D*—H	H···*A*	*D*···*A*	*D*—H···*A*
C2—H2···S1^i^	0.93	3.02	3.665 (3)	128
N5—H5*A*···N2	0.86 (1)	2.03 (3)	2.645 (4)	128 (3)
N5—H5*A*···O1	0.86 (1)	2.59 (2)	3.314 (4)	143 (3)
N5—H5*B*···N4^ii^	0.86 (1)	2.14 (1)	2.998 (4)	172 (3)
N6—H6*A*···O1^ii^	0.86 (1)	2.27 (2)	3.048 (4)	151 (4)
N6—H6*B*···O1^iii^	0.86 (1)	2.13 (1)	2.988 (4)	177 (4)
O1—H1*A*···N1	0.84 (1)	2.13 (3)	2.923 (4)	158 (6)
O1—H1*B*···N2^iv^	0.84 (1)	2.31 (2)	3.143 (4)	170 (9)

Symmetry codes: (i) *x*+1/2, −*y*+3/2, *z*−1/2; (ii) −*x*+1, −*y*+1, −*z*+1; (iii) *x*−1, *y*, *z*+1; (iv) *x*+1, *y*, *z*.

## References

[bb1] Apinitis, S. K. (1978). *J. Struct. Chem.* **19**, 158–161.

[bb2] Bruker (2016). *APEX3*, *SADABS* and *SAINT*. Bruker AXS Inc., Madison, Wisconsin, USA.

[bb3] Dolomanov, O. V., Bourhis, L. J., Gildea, R. J., Howard, J. A. K. & Puschmann, H. (2009). *J. Appl. Cryst.* **42**, 339–341.

[bb4] Groom, C. R., Bruno, I. J., Lightfoot, M. P. & Ward, S. C. (2016). *Acta Cryst.* B**72**, 171–179. 10.1107/S2052520616003954 PMC4822653 27048719

[bb5] Hens, A., Mondal, P. & Rajak, K. K. (2015). *Polyhedron*, **85**, 255–266.

[bb6] Kaim, W. (2001). *Coord. Chem. Rev.* **219–221**, 463–488.

[bb7] Kaur, B., Kaur, N. & Kumar, S. (2018). *Coord. Chem. Rev.* **358**, 13–69.

[bb8] Kurahashi, M. (1976). *Bull. Chem. Soc. Jpn*, **49**, 2927–2933.

[bb9] Kurahashi, M., Fukuyo, M., Shimada, A. & Kawase, A. (1976). *Bull. Chem. Soc. Jpn*, **49**, 872–875.

[bb10] Montelongo, F. G., Díaz, V. G. & González, C. R. T. (1982). *Microchem. J.* **27**, 194–199.

[bb11] Piyasaengthong, A., Boonyalai, N., Suramitr, S. & Songsasen, A. (2015). *Inorg. Chem. Commun.* **59**, 88–90.

[bb12] Sheldrick, G. M. (2015*a*). *Acta Cryst.* A**71**, 3–8.

[bb13] Sheldrick, G. M. (2015*b*). *Acta Cryst.* C**71**, 3–8.

[bb14] Zhang, G., Wang, S., Gan, Q., Zhang, Y., Yang, G., Shi Ma, J. & Xu, H. (2005). *Eur. J. Inorg. Chem.* pp. 4186–4192.

